# Secreted Extracellular Products of *Flavobacterium covae* as Potential Immunogenic Factors for Protection against Columnaris Disease in Channel Catfish (*Ictalurus punctatus*)

**DOI:** 10.3390/pathogens12060808

**Published:** 2023-06-07

**Authors:** Mohamed Sayed, Lakshmi Narayanan, Manal Essa, Mark Lawrence, Attila Karsi, Hossam Abdelhamed

**Affiliations:** 1 Department of Comparative Biomedical Sciences, College of Veterinary Medicine, Mississippi State University, Mississippi State, MS 39762, USAkarsi@cvm.msstate.edu (A.K.); 2 Department of Fish Diseases and Management, Faculty of Veterinary Medicine, Beni-Suef University, Beni-Suef 62511, Egypt; essa_manal@yahoo.com

**Keywords:** *Flavobacterium covae*, secreted extracellular proteins, protection

## Abstract

Columnaris disease caused by *Flavobacterium covae* leads to substantial economic losses in commercially important fish species worldwide. The US channel catfish (*Ictalurus punctatus*) industry is particularly vulnerable to this disease. Therefore, there is an urgent need to develop a vaccine to reduce the economic losses caused by this disease. Secreted extracellular products (SEPs) are considered to be essential bacterial virulence factors that often provide immunogenicity and protection. The current study sought to identify the main SEPs of *F. covae* and to evaluate their potential to provide protection in channel catfish against columnaris disease. SDS-PAGE analysis of SEPs revealed five protein bands with molecular weights ranging from 13 to 99 kDa. Mass spectrometry analysis showed that these SEPs were hypothetical protein (AWN65_11950), zinc-dependent metalloprotease (AWN65_10205), DNA/RNA endonuclease G (AWN65_02330), outer membrane protein beta-barrel domain (AWN65_12620), and chondroitin-sulfate-ABC endolyase/exolyase (AWN65_08505). Catfish fingerlings were vaccinated with SEPs, SEPs emulsified with mineral oil adjuvant, or heat-inactivated SEPs, or they were sham-immunized through intraperitoneal (IP) injection. After 21 days, an *F. covae* challenge showed 58.77% and 46.17% survival in the catfish vaccinated with the SEPs and the SEPs emulsified with adjuvant compared to the sham-vaccinated control (100% mortality within 120 h post-infection). However, the heat-inactivated SEPs failed to provide significant protection (23.15% survival). In conclusion, although SEPs contain potentially important immunogenic proteins, further work is needed to optimize their use for long-lasting protection against columnaris disease in fish. These results are significant given the economic impact of columnaris disease on fish farming worldwide.

## 1. Introduction

*Flavobacterium covae* (previously known as *F. columnare* genetic group 2) is a Gram-negative bacterium that causes columnaris disease in freshwater fish and poses a significant problem for aquaculture. A recent phylogenetic study has revealed that there are four distinct genetic groups within the genus *Flavobacterium* that represent four different species, all of which are the causes of columnaris disease [1]. In the United States, *F. covae* is a significant pathogen in the catfish industry, with mortality rates as high as 60% and 90% in adult and fingerling catfish, respectively [2]. The infection typically starts on the external mucosal surfaces of the fish and can cause yellow-orange lesions along the dorsal midline; this is referred to as saddleback disease [3,4]. The gills of the infected fish turn yellowish-white and can be damaged, leading to respiratory distress and death [3,5].

Outbreaks of columnaris disease usually occur during the production cycle’s spring and summer months under stressful environmental conditions [6]. Several authors demonstrated that columnaris disease outbreaks heavily depend on environmental and host-related factors [3]. Temperature fluctuation, higher feeding rates, high organic loads, high stocking density, oxygen deprivation, skin and gill damage, unnecessary handling, and poor water quality are the main factors that increase the risk of columnaris disease outbreaks [7,8]. In addition to environmental and host-related factors, the pathogenicity of *F. covae* isolates also affects the course of the disease, with different genetic groups expressing different degrees of virulence [9,10,11].

The control of columnaris disease outbreaks usually relies on antimicrobial agents, better husbandry/management practices to manage the stress level, vaccines, and water disinfection [3,12]. Over the past decades, a continuous effort has been made to develop a vaccine against *F. columnare* and *F. covae*. However, there have been some inconsistent results. Immersion immunization of carp (*Cyprinus carpio*) and channel catfish with formalin-killed bacterin resulted in a low level of protection [13]. A modified live rifampicin-resistant mutant of *F. covae* is currently registered for use in channel catfish, and it is also tested in largemouth bass (*Micropterus salmoides*) [5]. However, this vaccine is not widely used by farmers [14].

The pathogenesis of *F. columnare* and *F. covae* is complex and involves a variety of secreted extracellular proteins (SEPs) that facilitate colonization and survival in host tissues and induce a range of host immune responses. The extensive destruction of skin, muscle, and gill tissue in diseased fish may be partly explained by the production of various SEPs by *F. covae* [4,15]. The SEP profiles of virulent *F. covae* strains are different from those of the avirulent *F. covae* strains [16]. *Flavobacterium* species produce a range of SEPs, including proteases (metalloproteases, serine proteases, and cysteine proteases), chondroitin sulfate lyases, and hemolysin, which are considered important virulence factors. Proteases could mediate virulence through the degradation of host tissue and the evasion of host immune responses [17,18]. For example, metalloproteases cleave gelatin, casein, hemoglobin, fibrinogen, and elastin [15,17,18]. Chondroitin AC lyase is presumably responsible for the extensive necrotic lesions through the degradation of chondroitin sulfates (A and C) and the hyaluronic acid complex (polysaccharides of connective tissue) [19]. This enzyme acts specifically on a group of acidic mucopolysaccharides found primarily in animal connective tissue [15]. Indeed, a correlation was found between the degree of virulence and chondroitin AC lyase activity [9]. *F. covae* hemolysins can lyse red blood cells and other host cells, contributing to the pathogenesis of *F. covae* by promoting tissue damage and the evasion of host immune responses [20].

In this study, we extracted, concentrated, and identified the secreted extracellular proteins (SEPs) of *F. covae*. Our goal was to evaluate the potential of these proteins as a vaccine candidate for protecting channel catfish against columnaris disease. Identifying immunogenic or protective components in bacteria can serve as a basis for the development of potential vaccines against the pathogen.

## 2. Materials and Methods

### 2.1. Ethics Statement

The catfish experiments were completed according to the guidelines of an approved protocol by the Institutional Animal Care and Use Committee at Mississippi State University (IACUC-19–388).

### 2.2. Bacterial Strains and Culture Conditions

*F. covae* strain 94-081, originally isolated from channel catfish in the southeastern United States, was used in the present study [21,22]. The *F. covae* 94-081 strain was cultured at 28 °C with shaking in *F. covae* growth medium (FCGM) broth or agar (tryptone 8 g, yeast extract 0.8 g, MgSO_4_ 7H_2_O 1 g, CaCl_2_ 2H_2_O 0.74 g, NaCl 5 g, sodium citrate 1.5 g, and ddH_2_O 1 L) [23].

### 2.3. Preparation and Concentration of SEPs

The secreted extracellular proteins (SEPs) of the *F. covae* 94-081 strain were extracted with dialysis bags using a modification of a previously described procedure [24,25]. Briefly, the 94-081 strain was inoculated into FCGM broth and incubated for 3 h at 28 °C until the optical density at 600 nm (OD600) reached ~0.5. Then, cultures were used to inoculate (1:100 dilution) 250 mL of FCGM and were grown for 18 h at 28 °C. After incubation, the cultures were centrifugated (5000 rpm at 10 °C for 20 min). Then, the collected supernatant was passed through a syringe filter of 0.22 µm (VWR International, LLC, Radnor, PA, USA). A loopful of the filtrate was streaked on FCGM agar plates to ensure complete sterility. Proteins in the filtrate were concentrated eight times using dialysis tubing cellulose membrane (Sigma-Aldrich, St. Louis, MO, USA) with an average flat width of 25 mm and molecular weight cutoffs (MWCO) of 200 kDa. Afterwards, the resulting concentrated SEPs (5 mL) were dialyzed against phosphate buffer saline (PBS) solution with several buffer changes for 24 h at 4 °C to remove any traces of FCGM. Then, the obtained concentrated SEPs were kept at −20 °C until they were analyzed.

### 2.4. Determination of Protein Contents of SEPs

The total protein concentration of the SEPs was measured using Bradford’s method with Bradford Reagent (2-D Quant Kit-GE, Cytiva Marlborough, MA, USA) and bovine serum albumin (BSA) solution as a standard [26]. Samples of SEPs before and after dialysis were separated using sodium dodecyl sulfate-polyacrylamide gel electrophoresis (SDS-PAGE). A total of 20 µL of SEPs (5–10 μg/mL) was loaded on precast gels (Bio-Rad, Hercules, CA, USA) for a 12% Tricine SDS-PAGE. After electrophoresis, the gel was stained with 0.1% Coomassie Brilliant Blue (Bio-Rad, Hercules, CA, USA) for 45 min, followed by destaining. The PageRuler™ Prestained Protein Ladder, 10 to 180 kDa (Bio-Rad, Hercules, CA, USA), was included to estimate the molecular size of the protein bands.

Prior to mass spectrometric analysis, the gel bands were subjected to in-gel digestion using the In Gel Tryptic Digestion Kit^®^ (Thermo Scientific, Waltham, MA, USA). Briefly, individual gel bands were excised from the gel and cut into small pieces (2 mm in diameter). The gel pieces were washed three times by incubation for 30 min in a destaining solution (ammonium bicarbonate mixed with acetonitrile) at 37 °C with shaking. A reduction and alkylation process was performed to improve the quality of the obtained digest. To reduce the proteins, 3.3 µL of 50 mM Tris-2-carboxyethyl-phosphine (TCEP) mixed with digestion buffer was added to each sample and allowed to incubate at 60 °C for 10 min. After incubation, the reducing buffer was discarded from the tubes. Alkylation of the reduced proteins was performed by adding 25 μL of 400 mM iodoacetamide (IAA) and incubating the sample for 30 min at 37 °C with agitation in the dark. The reduction and alkylation process was repeated two times. The gel pieces were rewashed by incubation for 15 min at room temperature with acetonitrile. The acetonitrile was removed, and the gel pieces were dried for 5–10 min. Activated trypsin solution (~10 ng/µL) in digestion buffer was added to each sample followed by incubation at 37 °C for 4 h with shaking. The digestion mixture was removed and placed in a clean tube.

### 2.5. Preparation of Proteins Samples for Mass Spectrometry Analysis

Tryptic-digested peptides were desalted with C18-Ziptip using the protocol provided by the manufacturer (Millipore, Billerica, MA, USA). The hybrid quadrupole Orbitrap (Q Exactive Plus) MS system (Thermo Fisher Scientific, Bremen, Germany) was used in conjunction with an automated Easy-nLC 1200 system (Thermo Fisher Scientific, Bremen, Germany) for peptide separation. Peptides from three biological replicates were loaded onto an Acclaim Pepmap 100 pre-column (20 mm  ×  75 μm; 3 μm-C18) and separated on a PepMap RSLC analytical column (150 mm  ×  75 μm; 2 μm-C18) at a flow rate of 250 nL/min during a linear gradient from solvent A (0.1% formic acid in water) to 30% solvent B (0.1% formic acid, 19.9% water, and 80% acetonitrile) for 45 min and to 100% solvent B for an additional 15 min. The spectrum library was produced in the data-dependent mode with survey scans acquired at a resolution of 70,000 at 200 *m*/*z*. The mass spectrometer operated in MS/MS mode, scanning from 350 to 1600 *m*/*z*. Up to the top 10 most abundant isotope patterns with charges of 2~5 from the survey scan were selected with an isolation window of 1.2 Th and fragmented by high-energy collision dissociation (HCD) with normalized collision energies of 28. The maximum ion injection times for the survey and MS/MS scans were 250 ms, respectively, and the ion target values were set to 3E6 and 1e6, respectively. Selected sequenced ions were dynamically excluded for 30 s.

### 2.6. Data Search and Peptide Identification

Tandem mass spectra were extracted by Proteome Discoverer version 2.2 (Microsoft windows 7), and all the MS/MS samples were analyzed using Mascot (Matrix Science, London, UK; version 2.6.2). Mascot was set up to search the customized *Flavobacterium covae* 20200204 database (19,175 entries), assuming the digestion enzyme trypsin. Mascot was searched with a fragment tolerance of 0.01 Da and a mass tolerance of 10.0 PPM. O+18 of pyrrolysine and carbamidomethyl of cysteine were specified in Mascot as fixed modifications. Gln-pyro-Glu of the n-terminus, deamidation of asparagine and glutamine, and oxidation of methionine were specified in Mascot as variable modifications. Scaffold v. 4.2.1 (Proteome Software Inc., Portland, OR, USA) was used to validate MS/MS-based peptide and protein identifications. Peptide identifications were accepted if they could be established at greater than 95.0% probability by the Scaffold Local FDR algorithm. Protein probabilities were assigned by the Protein Prophet algorithm [27].

### 2.7. Fish Vaccination

Specific pathogen-free channel catfish fingerlings with a mean weight of 40.50 g (*n* = 200) were stocked into 20 flow-through 40 L tanks with continuous aeration at a density of 10 fish per tank. After a week of acclimation and being fed twice a day, the fish were randomly assigned to five groups with four replicate tanks in each group. The water temperature was maintained at 28 °C throughout the experiment.

The first group was intraperitoneally injected with 100 μL of SEPs at a concentration of 2.423 μg/µL [28]. The second group was injected with 100 μL of heat-inactivated (65 °C for 20 min) SEPs by the same method. The third group was injected with 100 μL of a mixture consisting of SEPs emulsified with Freund’s Complete Adjuvant (Seppic, Paris, France) at a ratio of 30:70 protein to adjuvant. The fourth group was sham-vaccinated with 100 μL of sterile PBS. The fifth group served as the negative control (sham-sham).

Before handling, the fish were anesthetized with tricaine methanesulfonate (MS-222; Sigma^®^). At day 21 post-immunization, all the fish were experimentally infected with *F. covae* strain 94-081 by immersing them in water containing 2.56 × 10^7^ CFU/mL of bacteria for 2 h [21,29]. The bacterial infection dose was chosen based on previous experimental infection doses. *F. covae* typically affects the gills, the skin, and fins [3]. Thus, the challenge by immersion is closer to the natural route of infection [30]. Mortalities were recorded daily for 2 weeks in each group to calculate the percent survival and to assess the protective effect of the vaccination. *F. covae* was confirmed as the cause of death by culturing gills swabs on FCGM and incubating at 28 °C.

### 2.8. Statistical Analysis

The mortality data obtained from the different vaccination groups were arcsine transformed, and analysis of variance (ANOVA) was applied using PROC GLM in SAS for Windows v9.4 (SAS Institute, Inc., Cary, NC, USA) to determine significance. The statistical significance level was set at alpha = 0.05 for all the analyses.

## 3. Results

### 3.1. SEP Profiles

**Figure 1:**
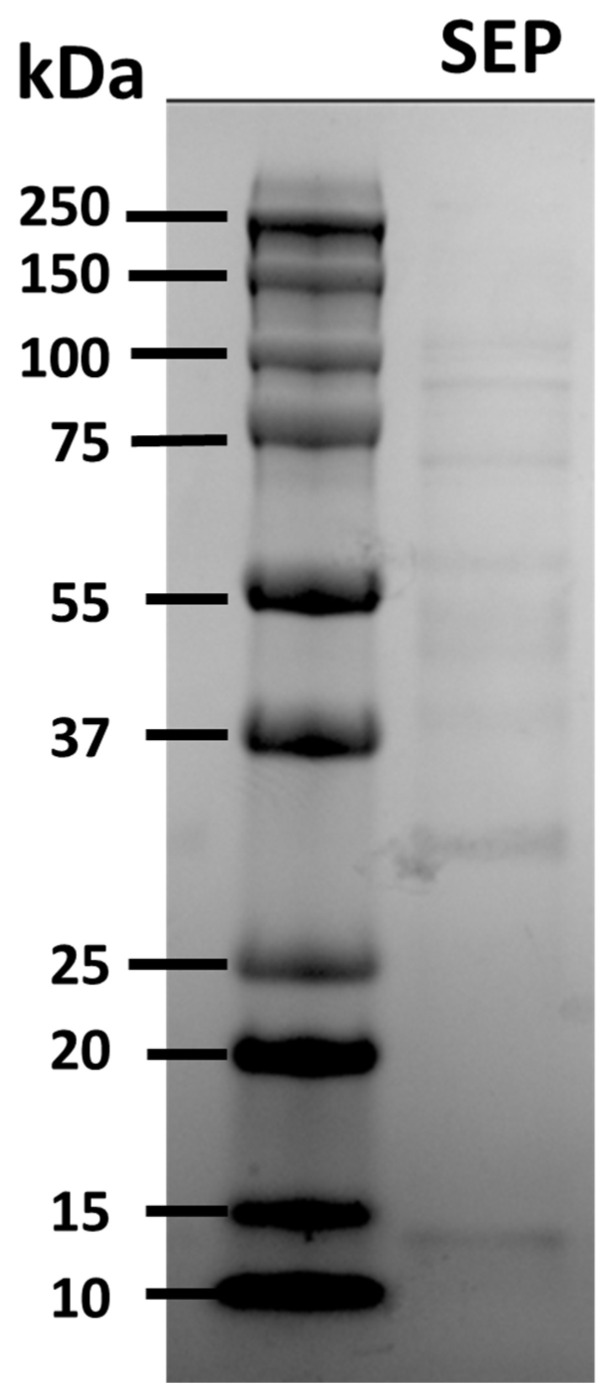
SDS-PAGE with Coomassie blue stain showing purified SEPs of *F. covae* 94-081 grown in FCGM. Molecular weights in kilodaltons (kDa) are shown for the standard protein marker in the right column.

The SEPs were prepared and demonstrated to be free of *F. covae* contamination by culturing on FCGM plates. The concentrated proteins from the SEPs were visualized on SDS-PAGE (Figure 1), revealing protein bands with molecular weights ranging from 13 to 99 kDa.

### 3.2. Mass Spectrometric Analysis

Our findings revealed that the 13 kDa band exhibited a high degree of similarity to a hypothetical protein (AWN65_11950), while the 30 kDa band was identified as a zinc-dependent metalloprotease (AWN65_10205). The 34 kDa band was identified as a DNA/RNA endonuclease G protein (AWN65_02330), and the 35 kDa band was identified as an outer membrane protein beta-barrel domain (AWN65_12620). Furthermore, the 99 kDa band showed a high degree of similarity to a glycosaminoglycan (GAG) polysaccharide lyase or chondroitin-sulfate-ABC endolyase/exolyase (AWN65_08505). For a summary of the putative functions of these identified proteins, please refer to Table 1.

### 3.3. Vaccination Efficacy

**Figure 2:**
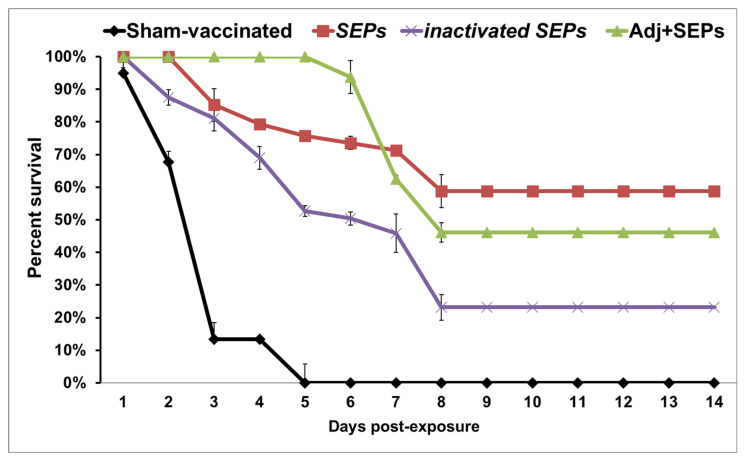
Percent survival of channel catfish fingerlings immunized with the SEPs, heat-inactivated SEPs, or SEPs emulsified with adjuvant (Adj + SEPs) and challenged with *F. covae* 94-081 strain at 21 days post-exposure. Data are presented as mean ± SE of four replicate tanks.

The survival percentages of the catfish fingerlings vaccinated with SEPs, SEPs emulsified with adjuvant, and heat-inactivated SEPs after 21 days post-infection were 58.77%, 46.17%, and 23.15%, respectively (Figure 2). These results indicate a significantly higher survival rate (*p* < 0.05) for the catfish immunized with SEPs and SEPs emulsified with adjuvant compared to that of the sham-vaccinated group, which had 0% survival. There was no significant difference in survival between the heat-inactivated SEP vaccine group and the sham-vaccinated control. Notably, mortality progressed slower in the catfish vaccinated with SEPs emulsified with adjuvant (starting at 144 h post-infection) than in the sham-vaccinated group, which had 100% mortality within 120 h post-infection. All the dead fish had external lesions which were consistent with those of *F. covae* infection. *F. covae* was isolated from the gills of the dying fish to confirm the cause of death.

## 4. Discussion

Vaccines are critical in managing infectious diseases in animals, including fish. This study aimed to explore the potential of *F. covae* SEPs as vaccine candidates against virulent *F. covae*. SEPs, which are secreted by Gram-negative pathogens to manipulate or damage host cells [31], are important in the pathogenesis of several fish pathogens and provide sufficient immunogenicity to confer protection against infection following immunization. Our rationale for evaluating SEPs was based on the findings of previous studies in which SEP antigens were able to stimulate an immune response and provide immune protection against piscine pathogens such as *Photobacterium damselae* spp. [32], *Vibrio harveyi* [33], *Flavobacterium psychrophilum* [34], and *F. covae* [35]. The immunogenic and protective efficacy of SEP vaccines can be improved by concentrating specific antigens. In this study, we used the highly virulent *F. covae* strain 94-081 to prepare SEPs.

The pathogenesis of *F. covae* involves the secretion of extracellular products and enzymes that play important roles in the adhesion and lysis of skin, muscle, and gill tissues. [15]. In the present study, five proteins with molecular weights ranging from 13 to 99 kDa were observed in the SEPs from FCGM supernatant. The proteins were identified using MS/MS as zinc-dependent metalloprotease, DNA/RNA endonuclease G, outer membrane protein beta-barrel domain, chondroitin-sulfate-ABC endolyase/exolyase, and hypothetical protein. These five secreted proteins might be potential virulence factors facilitating host tissue adhesion or cellular lysis. Other studies have identified protein antigens in *Flavobacterium.* For example, five outer membrane proteins of *F. covae*, including zinc metalloprotease, prolyl oligopeptidase, thermolysin, collagenase, and chondroitin AC lyase were detected using recombinant fusion protein [28]. In addition, four immunogenic protein bands were found from the OMPs of *F. covae* by SDS-PAGE and Western blotting using antisera obtained from mandarin fish (*Siniperca chuatsi*) [36]. Using 2-DE gels, 15 immunogenic proteins from the cellular and extracellular products of a virulent *F psychrophilum* strain and 4 from a non-virulent strain were identified [37].

Among the proteins identified in SEPs, one band with an approximate molecular weight of 99 kDa was observed that encodes chondroitin-sulfate-ABC endolyase/exolyase. It has been reported that *F. covae* secretes chondroitin AC lyase, which is responsible for the degradation of chondroitin sulfate. Previous studies suggested that chondroitin AC lyase may play an essential role in the severity of *F. covae* invasion, necrosis, and tissue destruction [18]. Moreover, it has been demonstrated that polysaccharide lyase activity is strongly related to the virulence of *F. covae* [11]. Additionally, it was shown that highly virulent *F. covae* strains (rhizoid) produce higher polysaccharide lyase activity than low-virulence variants (rough) of the same strains [9].

Several secreted zinc-dependent metalloproteases were predicted in the *F. covae* genome [20]. This study identified a 35 kDa zinc-dependent metalloprotease in SEPs of *F. covae*. Zinc metalloprotease is considered a virulence factor in several fish pathogens, including *F. covae* [19], *Vibrio anguillarum* [38], *Yersinia ruckeri* [39], and *Aeromonas hydrophila* [40]. The strong proteolytic activity of *F. covae* and *F. psychrophilum* species indicates that the production of extracellular metalloproteases is an important component of virulence that promotes the invasion and hydrolysis of the fish muscular proteins [19,41].

An outer membrane protein (OMP) with a beta-barrel domain was detected in the SEPs of *F. covae*. OMPs have been found to play crucial roles in essential transport and protective functions, including binding and delivering DNA, transporting virulence factors, defense against antimicrobials, and removing toxic envelope proteins [42]. OMPs produced by Gram-negative bacteria have been identified as promising candidates for vaccine development due to their presence on the bacterial cell surface, which allows them to be easily recognized by the host immune system [43,44]. It has been reported that immunization with recombinant OMPs can induce protective immunity in host species from pathogenic bacteria such as *Vibrio parahaemolyticus* [45], *A. hydrophila* [46], *Streptococcus iniae* [47], and *Edwardsiella tarda* [48]. Components of OMPs in *F*. *covae* were capable of evoking an immune response and providing immune protection against *F. covae* infection in Bluntnose black bream (*Megalobrama amblycephala*) and in channel catfish [35,49].

In addition, the present study identified a small protein with a molecular weight of approximately 13 kDa in the SEPs. This protein is currently designated as a hypothetical protein of *F. covae*, as it has no known function or identity with any characterized proteins. Previous studies on *F. covae* have noted a higher abundance of this small protein in virulent strains compared to that in non-virulent strains [16]. This suggests that the protein may play a role in the virulence of *F. covae*, although its specific function remains unknown. We also identified endonuclease in the SEPs of *F. covae*. Extracellular endonucleases are known to have a role in the virulence of several bacterial species, where they mediate phenotypes such as biofilm formation, the degradation of neutrophil extracellular traps, and the degradation of host genomic DNA as a nutrient source [50,51]. It is important to emphasize that one-dimensional gel electrophoresis separates protein only based on the molecular weight, whereas two-dimensional gel electrophoresis segregates proteins on the basis of molecular weight and the isoelectric point.

The present study showed that SEPs were safe for administration by injection, and a single dose of SEPs or SEPs emulsified with adjuvant provided significant protection in catfish against the *F. covae* 94-081 strain. The observed protection is likely due to specific immunity stimulated by antigens in the SEPs, but non-specific immune stimulation may also have contributed. Further study will focus on the expression and purification of these proteins and evaluate the potential immunogenicity of each recombinant protein. While the level of protection achieved in our study was moderate (58% survival rate), this result is still significant from an economic standpoint considering the high value of channel catfish, the severity of columnaris disease at fish farms, and the lack of preventive methods. The moderate percent survival observed in our study could be due to the high *F. covae* infection dose (2.25 × 10^7^ CFU/mL of water) or the small size of the fish used in the experiment. In a previous study, catfish vaccinated with a single immersion vaccination with monovalent modified *F. columnare* vaccine demonstrated relative percent survival (RPS) values of 50.0%, 57.0%, and 76.8% at days 109, 116, and 137 post-primary immunization, respectively, when challenged with *F. columnare*, while the fish that received a booster immunization with the rifampicin modified *F. columnare* vaccine at day 34 exhibited RPS values of 64.8% and 65.2% at days 109 and 137 post-primary immunization after being challenged with *F. columnare* [52].

Notably, the mortalities of the non-vaccinated fish infected with *F. covae* strain 94-081 reached 100% within 120 h post-infection. To further investigate the efficacy of SEP vaccination, future research will evaluate the protection level against lower challenge doses and in larger catfish. Additionally, administering a booster dose and using immunopotentiators as prophylactic adjuvants could potentially improve the effectiveness of SEP vaccination. Future studies will also investigate the efficacy of various vaccine delivery routes and determine whether there is cross-protection against a range of *F. covae* isolates.

In conclusion, our study identified five proteins with molecular weights ranging from 13 to 99 kDa in the SEPs of *F. covae*; these could potentially be virulence factors. However, their precise functions require further elucidation. Our immunization trial showed that administering SEPs provided significant protection in catfish against virulent *F. covae* infection, indicating that the SEPs have the potential to be protective antigens in vaccination against columnaris disease.

## Figures and Tables

**Table 1:** Extracellular proteins of *F. covae* 94-081 by MS/MS analysis, and the subsequent identification by BLAST search. ORF annotation refers to *F. covae* 94-081 complete genome [NCBI: NZ_CP013992.1].

Locus Tag	Size (kDa)	Protein Name	Accession Number in NCBI
AWN65_08505	99	Glycosaminoglycan (GAG) polysaccharide lyase	AMA49498.1
AWN65_10205	30	Zinc-dependent metalloprotease	AMA49793.1
AWN65_02330	34	DNA/RNA Endonuclease G	AMA48388.1
AWN65_12620	35	Outer membrane beta-barrel protein	AMA50710.1
AWN65_11950	13	Hypothetical protein	AMA50124.1

## Data Availability

The data that support the findings of this study are available on request from corresponding author.
